# Association between COVID-19 stress, coping mechanisms and stress-related oral conditions among Egyptian adults: a cross-sectional study

**DOI:** 10.1038/s41598-022-22961-z

**Published:** 2022-10-27

**Authors:** Nourhan M. Aly, Amira H. Elwan, Raghda M. Elzayet, Nour M. R. Hassanato, Mariam Deif, Wafaa E. Abdelaziz, Maha El Tantawi

**Affiliations:** Department of Pediatric Dentistry and Dental Public Health, Faculty of Dentistry, Alexandria University, Champolion St., Azarita, Alexandria, 21527 Egypt

**Keywords:** Dentistry, Public health

## Abstract

The present study investigated the association between COVID-19 stresses and oral conditions including gingivitis, oral hygiene, oral ulcers, and dry mouth. This was a cross-sectional study that collected data from adults in community settings in Alexandria, Egypt, between October 2021, and February 2022. Gingival condition and oral hygiene were assessed using the gingival and plaque indices. Participants were asked if they experienced oral ulcers during the past week and dry mouth during the past year. COVID-19 fears and coping were assessed using the COVID Stress Scale (CSS), and the Brief Resilience Coping Scale (BRCS), respectively. Oral health behaviors were assessed using the World Health Organization questionnaire. Regression analyses were used to assess the association between the dependent variables (clinically assessed gingival and plaque indices, reported presence of oral ulcers, and dry mouth) and explanatory variables (CSS and BRCS) after adjusting for confounders (COVID-19 status, oral health behaviors, smoking, age in years, sex, and highest educational level). The response rate was 88.8% (373/420). The mean (SD) age = 39.26 (11.45) with 74.3% females and 49.3% reporting completing high school or higher education. The mean (SD) plaque and gingival indices were 1.59 (0.66) and 1.39 (0.59), respectively. Only 20.1% reported the presence of oral ulcers and 41.6% reported xerostomia. Lower plaque score was associated with higher COVID-19 contamination fears (B = − 0.03, 95% CI − 0.05, − 0.02) and higher compulsive checking and reassurance-seeking (B = − 0.02, 95% CI − 0.03, − 0.009). Lower gingival score was associated with higher COVID-19 contamination fears (B = − 0.02, 95% CI − 0.03, − 0.002). Higher odds of reporting dry mouth were associated with greater fear of COVID-19 socioeconomic consequences (AOR = 1.05, 95% CI 1.001, 1.09), and lower coping scores (AOR = 0.93, 95% CI 0.88, 0.99). The findings suggest an association between COVID-19 specific stresses and stress-related oral conditions and shed light on the possible link between mental and oral health, emphasizing the importance of integrated planning of care services.

## Introduction

In 2019, the world was hit by the COVID-19 pandemic which caused a significant impact on physical and mental health^1^ including anxiety, depression, and stress. In addition, insomnia, dissatisfaction, phobias, compulsive behaviors, physical symptoms, and social functioning impairment were reported during the pandemic^2^. Research assessing COVID-19 effects showed a significant psychological impact across the globe with many reported disturbances including post-traumatic stress disorder, and depression^3^.

Research showed a link between COVID-19 infection and oral health conditions^4–6^ with adverse oral health manifestations including taste disorders, aphthous-like lesions, herpetiform lesions, candidiasis and desquamative gingivitis^6–8^. Also, COVID-19 infection was reported to have direct effects on salivary glands causing xerostomia which may further lead to dental caries, fissuring of the lips and oral mucosa, ulcerations, and inflammation of the oral mucosa and tongue^9^.

On the other hand, several studies reported an association between stress, anxiety and depression on one hand, and oral health conditions such as erosion, caries, and periodontal disease on the other hand^10,11^. Depression was also associated with decreased utilization of dental services^11,12^. Moreover, depression may drive people to adopt unhealthy practices to cope with stresses such as high consumption of refined carbohydrates, use of psychoactive substances, tobacco, and alcohol and these are reported to increase the likelihood of developing oral diseases^12,13^. Chronic stress can also contribute to the dysfunction of physiologic systems and may affect disease progression in case of periodontal disease^13^, recurrent oral ulcerations, burning, and dry mouth^14^. Evidence also showed associations between stress and poor self-rated oral health and oral health-related quality of life^15,16^.

In addition to the potential biologic mechanisms for the effect of COVID-19 infection on oral health^17,18^, part of the adverse effects may be attributed to COVID-19 specific stresses. Perception of stress is related to a specific event, several events, or an accumulation of events and is not a trait. It manifests as a response to an incident rather than individual differences in reactions^19^. The COVID-19 pandemic is a stressor of unprecedented scale and impact. Thus, it is important to assess stresses caused by the pandemic using specific tools that can capture the event-related impact as opposed to chronic or lifetime stresses assessed during the pandemic. The COVID Stress Scales (CSS)^20^ assesses the different stresses caused by the COVID-19 pandemic and allows the quantification of its impact in distinction from general stresses caused by other factors. This assessment helps in understanding the mechanisms by which COVID-19 affects oral health.

To our knowledge, few studies explored the association between COVID-19 stress, coping mechanisms and oral health outcomes. Thus, the aim of the present study was to investigate whether COVID-19 stresses were associated with oral conditions such as gingival inflammation, poor oral hygiene, oral ulcers, and xerostomia among adults in Egypt. The null hypothesis of the study was that there would be no association between COVID-19 stresses and oral health conditions.

## Methods

### Design

This was a cross-sectional study that collected data from adults in community settings in Alexandria, Egypt, between October 2021 and February 2022. Ethical approval for the study was obtained from the Research Ethics Committee of the Faculty of Dentistry, Alexandria University, Egypt (IRB 00010556–IORG 0008839).

### Participants and sampling

A random sample of adults was recruited from all administrative districts of Alexandria during medical convoys organized by Alexandria University as part of its outreach program to serve the community during the COVID-19 pandemic. The convoys targeted underserved populations in various administrative districts in the governorate. The population reached was mainly of modest socio-economic background. During these convoys, local guides helped in the random selection of participants living in the region and they gathered in a school, a charity organization, a community leader’s house or a similar setting for clinical examination and questionnaire administration. Participants received treatment on site or were referred to the university clinics. For the present study, individuals aged 18 years or above, of both sexes, were invited to participate after obtaining their informed consent. Those who had major psychiatric disorders, uncontrolled autoimmune or metabolic diseases, or cognitive impairment were excluded.

### Sample size estimation

Sample size was based on 95% confidence level to detect a gingival inflammation level similar to that reported in a previous study^21^ conducted among Egyptian adults (mean gingival index = 1.66, SD = 0.44, calculated 95% confidence interval = 1.620, 1.702). The required sample size was 369 participants, calculated using MedCalc Statistical Software version 19.0.5 (MedCalc Software bvba, Ostend, Belgium; https://www.medcalc.org; 2019).

### Data collection

Data were collected through clinical examination and interview-based questionnaires. The questionnaire was uploaded on an online platform (KoboToolbox) that allowed offline data entry with subsequent synchronization when there was internet access. The questionnaire was preceded by a brief introduction explaining the purpose of the study, assuring the confidentiality of participants’ responses, and emphasizing that their participation was voluntary. This introduction was read by a researcher before the participant was interviewed. The questionnaires were pilot-tested at the Faculty of Dentistry, Alexandria University, Egypt. Data of the pilot-testing were not included in the final analysis.

### Dependent variables

1. Gingival inflammation and oral hygiene: The gingival condition was clinically assessed using the gingival index (GI) of Löe and Silness^22^, whereas oral hygiene was assessed using the plaque index (PLI) of Silness and Löe^23^. Both indices used the same four surfaces (buccal, lingual, mesial, and distal) on six index teeth (#16, #12, #24, #36, #32 and #44) and were scored from zero to 3. The scores were averaged to give tooth scores and all teeth scores were averaged to give an individual’s score. The GI index measures the severity of gingival inflammation, while the PLI measures the thickness of plaque in the gingival third of the tooth. Mirrors and ball ended World Health Organization (WHO) probes (#550B) were used for examination. Clinical examination was performed by two trained examiners after calibration using 20 intraoral photographs. Intra- and inter-examiner reliability were calculated using Kappa statistic which ranged from 0.82 to 0.88 indicating excellent agreement^24^. Calibration on patients could not be performed since the examination by one examiner could change the condition that subsequent examiners aimed to assess due to plaque removal or induction of gingival bleeding^25^.
2. Self-reported oral ulcers: Participants were asked if they experienced any oral ulcers during the past week (yes/no)^12^.
3. Self-reported xerostomia: Participants were asked if they experienced dry mouth during the past 12 months (yes/no)^26^.

### Independent variables

1. COVID-19 specific stresses: These were assessed using the CSS^20^. The CSS is composed of six subscales assessing different COVID-related stresses: (1) Danger fears (2) contamination fears, (3) fears about socioeconomic consequences, (4) xenophobia, (5) compulsive checking and reassurance-seeking, and (6) traumatic stress symptoms about COVID-19. The scale consists of 36 items scored on a five-point Likert scale ranging from 0 (not at all/never) to 4 (extremely/almost always). Participants were asked if they had experienced any of these worries during the past week. The total score of each domain was calculated by adding the scores of its items and higher scores indicated greater levels of COVID-19-specific stress. The score of each domain ranged from 0 to 24 and the total CSS score ranged from 0 to 144. The scale was originally developed and validated in English^20^ and further translated and validated in Arabic^27^. Cronbach’s alpha for the internal consistency of the items in this study was 0.95. The scores were used as quantitative variables.
2. Coping: This was assessed using the Brief Resilience Coping Scale (BRCS)^28^ which assessed the ability to cope with stressful situations. The scale is composed of four questions rated on a five-point Likert scale ranging from 1 (does not describe me at all) to 5 (describes me very well). It was originally developed in English, then translated and validated in Arabic^29^. The total score is the sum of scores of the four questions and ranges from 0 to 20. Higher scores indicated better resilience and coping abilities. The Cronbach’s alpha for the scale in this study was 0.84. This score was, also, used in its quantitative form.

### Confounders

1. COVID-19 status: Participants were asked if they had previously tested positive for COVID-19, had a close friend who previously tested positive for COVID-19 and if they knew someone close who died from COVID-19^30^.
2. Oral health related behaviors: Oral health behaviors were assessed using the WHO questionnaire- adult form^26^ which was translated into Arabic language in a previous study^31^. Participants were asked about the frequency of toothbrushing (categorized for analysis into at least once daily versus less) and dental visits during the past 12 months (categorized for analysis into at least once versus less). Participants were also asked about their daily consumption of eight sugary and carbohydrate-containing items. A total sugar consumption score was calculated by adding the number of items consumed on daily basis. The sugar score ranged from 0 to 8. Higher scores indicated greater daily sugar consumption^32^.
3. Smoking: Participants were asked about their smoking status (never smoked, former smoker and current smoker) and responses were categorized into current smokers (yes/no)^26^.
4. Sociodemographic profile: including age in years, sex (male or female), highest educational level (non-educated, completed primary/middle school or completed high school/higher education).

### Statistical analysis

Data were analyzed using IBM SPSS for Windows (Version 23.0, IBM Corp., Armonk, N.Y., USA). Descriptive statistics were calculated as means, standard deviations (for quantitative variables), frequencies, and percentages (for qualitative variables). Two multivariable linear regression models were constructed where the dependent variables were plaque and gingival indices, and two binary logistic regression models were used where the dependent variables were self-reported presence of oral ulcers and xerostomia. All models were adjusted for potential confounders including COVID-19 status, oral health-related behaviors, smoking and the sociodemographic profile of participants (age in years, sex, and highest educational level). We calculated the regression coefficients (B), adjusted odds ratio (AOR), 95% confidence intervals (CI), adjusted R^3^ and Nagelkerke’s R^2^. Significance was inferred at p value < 0.05. Sensitivity analysis was conducted to assess robustness of the significant associations between dependent variables and each stress subscore to unmeasured confounders by calculating E-values (https://www.evalue-calculator.com/evalue/). The E-value is the minimum strength of an association between an unmeasured confounder and each of the dependent and independent variables that would explain away their significant association. Whereas no cut-off points are reported in the literature for E-values, large values indicate the robustness of the significant association between variables to the effect of unmeasured confounders, whereas small E-values indicate that little unmeasured confounders may explain away the observed significant associations^33^.

### Ethics declarations

Ethical approval was obtained from the Research Ethics Committee, Faculty of Dentistry, Alexandria University, Egypt (IRB 00010556–IORG 0008839) and was performed in full accordance with the Helsinki declaration. Informed consent was obtained from all participants.

## Results

Four hundred and twenty participants were invited, but only 373 agreed to participate (response rate = 88.8%). Table 1 shows the main characteristics of the study sample. Most participants were females (74.3%), the mean (SD) age = 39.26 (11.45) and 49.3% completed high school or higher education. Also, 41.6% reported xerostomia and 20.1% reported oral ulcers. Participants had moderate amount of plaque accumulation and gingival inflammation [mean (SD) index scores = 1.59 (0.66) and 1.39 (0.59), respectively]. The mean (SD) CSS was 55.81 (26.41), and the danger and contamination fears sub-domains had the highest scores [mean (SD) = 13.63 (5.91) and 11.21 (5.89), respectively]. The mean (SD) coping score was 13.52 (3.78).

**Table 1:** Sample description (n = 373).

Factors			N (%)
Age		Mean (SD)	39.26 (11.45)
Gender: n (%)		Male	96 (25.7%)
		Female	277 (74.3%)
Highest educational level: n (%)		Non-educated/less than primary	77 (20.6%)
		Completed primary/middle school	112 (30%)
		Completed high school/higher education	184 (49.3%)
Toothbrushing: n (%)		At least once daily	152 (40.8%)
		Less than once daily	221 (59.2%)
Dental visits during the past 12 months: n (%)		At least once	166 (44.5%)
		Less than once/never	207 (55.5%)
Xerostomia: n (%)		Yes	155 (41.6%)
		No	218 (58.4%)
Smoking: n (%)		Yes	50 (13.4%)
		No	323 (86.6%)
Tested COVID-19 positive: n (%)		Yes	56 (15%)
		No	317 (85%)
Had a close friend/relative tested COVID-19 positive: n (%)		Yes	253 (67.8%)
		No	120 (32.2%)
Knew someone who died because of COVID-19: n (%)		Yes	207 (55.5%)
		No	166 (44.5%)
Had oral ulcers in the past week: n (%)		Yes	75 (20.1%)
		No	298 (79.9%)
COVID Stress Scale (CSS)	Danger	Mean (SD)	13.63 (5.91)
	Contamination		11.21 (5.89)
	Socioeconomic consequences		9.65 (6.69)
	Xenophobia		9.73 (6.51)
	Traumatic stress		3.76 (5.26)
	Compulsive checking and reassurance-seeking		7.82 (6.48)
	Total score		**55.81 (26.41)**
Coping score			13.52 (3.78)
Sugar consumption score			2.23 (1.60)
Plaque index			1.59 (0.66)
Gingival index			1.39 (0.59)

*SD* Standard deviation.

Table 2 shows the association of stresses and coping score with plaque and gingival indices after controlling for confounders. Individuals with high COVID-19 contamination fears had significantly lower plaque and gingival indices (B = − 0.03, 95% CI − 0.05, − 0.02 and B = − 0.02, 95% CI − 0.03, − 0.002, respectively). Those with high compulsive checking and reassurance-seeking score had significantly lower plaque index (B = − 0.02, 95% CI − 0.03, − 0.009). The models explained 18% of the variation in the plaque index and 12% of the variation in the gingival index. The E values of the CSS sub-scores for contamination and compulsive checking in the plaque and gingival indices models ranged from 1.20 to 1.25. This indicates that the unmeasured confounders would need to have a strong association with the variables to explain away the observed associations. Specifically, this strong association is about one point change on a scale from 0 to 3 for both indices, signifying the robustness of both models to unmeasured confounders.

**Table 2:** Association of COVID-19 specific stresses and coping with plaque and gingival indices in adjusted multivariable regression.

Factors	Plaque index		Gingival index	
	B (95% CI)	P value	B (95% CI)	P value
CSS-danger subscore	0.009 (− 0.007, 0.03)	0.26	0.006 (− 0.009, 0.02)	0.43
CSS-contamination subscore	− 0.03 (− 0.05, − 0.02)	< 0.001*	− 0.02 (− 0.03, − 0.002)	0.02*
CSS-socioeconomic consequences subscore	− 0.008 (− 0.02, 0.004)	0.20	0.003 (− 0.008, 0.01)	0.58
CSS-xenophobia subscore	0.01 (− 0.002, 0.02)	0.09	0.005 (− 0.006, 0.02)	0.37
CSS-traumatic stress subscore	0.009 (− 0.005, 0.02)	0.21	0.006 (− 0.008, 0.02)	0.41
CSS-compulsive checking and reassurance-seeking subscore	− 0.02 (− 0.03, − 0.009)	0.001*	− 0.008 (− 0.02, 0.002)	0.12
Coping score	− 0.001 (− 0.02, 0.02)	0.87	0.001 (− 0.02, 0.02)	0.90
Model F (p value) Adjusted R^2^	5.17 (< 0.001*) 0.18		3.69 (< 0.001*) 0.12	

*B* Adjusted regression coefficient, *CI* confidence interval.

Both models were adjusted for age in years, sex, and highest educational level, history of COVID-19 infection, having a close friend testing COVID-19 positive, knowing someone who died of COVID-19, xerostomia, smoking, toothbrushing, dental visits, and sugar consumption.

*Statistically significant at p value < 0.05.

For the CSS- Contamination subscore, the E-value of the plaque index model = 1.25 and of the gingival index model = 1.21.

For the CSS-compulsive checking and reassurance-seeking subscore, the E-value of the plaque index model = 1.20.

Table 3 shows the association of stresses and coping score with reported presence of oral ulcers and xerostomia after controlling for confounders. Participants with greater fears of the COVID-19 socioeconomic consequences had significantly higher odds of reporting xerostomia (AOR = 1.05, 95% CI 1.001, 1.09). Participants with higher coping score had significantly lower odds of reporting xerostomia (AOR = 0.93, 95% CI 0.88, 0.99). The models explained 9% and 12% of the variation in reported presence of oral ulcers and xerostomia, respectively. The E-values of the CSS sub-scores for the socioeconomic consequences and coping score in the xerostomia model were 1.18 and 1.23. Thus, the unmeasured confounders would need to have a weak association with the variables to explain away the observed associations since the values were very close to the OR null value = 1. This indicates that the model is not robust to the effect of unmeasured confounders.

**Table 3:** Association of COVID-19 specific stresses and coping with reported oral ulcers and xerostomia.

Variables	Oral ulcers		Xerostomia	
	AOR (95% CI)	P value	AOR (95% CI)	P value
CSS-danger	1.03 (0.96, 1.10)	0.45	0.996 (0.94, 1.06)	0.89
CSS-contamination	1.00 (0.93, 1.07)	0.96	0.95 (0.89, 1.001)	0.054
CSS-socioeconomic consequences	0.97 (0.92, 1.02)	0.22	1.05 (1.001, 1.09)	0.047*
CSS-xenophobia	1.05 (1.00, 1.11)	0.06	0.98 (0.94, 1.03)	0.40
CSS-traumatic stress	1.01 (0.95, 1.07)	0.85	1.04 (0.99, 1.09)	0.16
CSS-compulsive checking and reassurance-seeking	1.01 (0.96, 1.06)	0.70	1.01 (0.97, 1.05)	0.60
Coping score	0.97 (0.90, 1.04)	0.38	0.93 (0.88, 0.99)	0.03*
Model X^2^ (p value)	5.13 (0.02*)		0.24 (0.63)	
Nagelkerke’s R^2^	0.09		0.12	

*AOR* Adjusted odds ratio, *CI* confidence interval.

*Statistically significant at p value < 0.05.

Both models were adjusted for age in years, sex, and highest educational level, history of COVID-19 infection, having a close friend testing COVID-19 positive, knowing someone who died of COVID-19, smoking, toothbrushing, dental visits, and sugar consumption. The model for oral ulcers was additionally adjusted for xerostomia.

In the xerostomia model, the E value of the CSS- Socioeconomic consequences subscore = 1.18 and the E-value of the coping score = 1.23.

## Discussion

The study showed that among a group of adult Egyptians, there were moderate levels of gingival inflammation and plaque accumulation. Also, about 20% and 40% respectively reported xerostomia and oral ulcers. The most frequently expressed COVID-19 stress was fear of danger and of contamination. The overall COVID stresses were below average, while coping scores were above average. Fear of COVID-19 contamination was associated with lower scores of plaque and gingival indices, whereas compulsive checking and reassurance-seeking were associated with less plaque accumulation. Stressing about the socioeconomic consequences of COVID-19 was associated with higher odds of reporting xerostomia, while greater coping and resilience were associated with lower odds of reporting xerostomia. Unmeasured confounders might have a greater role in explaining away the association between COVID-19 stress and xerostomia than between stress and plaque accumulation and gingivitis. Overall, COVID-19 stresses were associated with clinically assessed and patient reported oral health outcomes and the null hypothesis can be partially rejected.

The study has several strengths. First, we assessed COVID-19 specific stresses using a validated tool that captured multiple stresses specific to the pandemic. Second, we depended on clinical examination to assess oral conditions that may be affected by COVID-19 stresses and complemented our assessment by measuring patient-reported outcomes to comprehensively capture the impact of the pandemic on oral health. We assessed patient-reported outcomes using interview-based questionnaires. This allowed the inclusion of non-educated participants and resulted in a higher response rate and greater generalizability than if an online questionnaire would have been used^34^. Third, the population included in the study had higher than average level of resilience and below average COVID-19 stresses which shed light on the impact of COVID-19 on oral health in participants with a psychological profile different from that reported in the literature. Our study, thus, fills a knowledge gap by providing evidence about the association between mental and oral health in a developing country with different levels of stresses and resilience adding to the literature which comes from mostly developed countries.

However, the study had some limitations. First, its cross-sectional design cannot confirm causality and can only suggest associations. Because of this, future longitudinal studies are needed to assess the long-term impact of COVID-19 stress on oral health. Second, there was a potential risk of social desirability bias which may have resulted in over- or under-reporting of self-perceived COVID-19 fears, toothbrushing frequency, and sugar consumption. We tried to reduce this bias by using standardized and validated questionnaires. Also, measuring xerostomia using a single-item instead of a multi-component measure such as the xerostomia inventory scale might have affected the results. Third, some potential confounders may have not been explicitly assessed. However, we calculated the E-values to assess robustness of the models to the effect of unmeasured confounders so that readers can make informed decisions. Vaccination is assumed to decrease COVID-19 related stress^35^ and it should be assessed in future studies addressing COVID-19 stress.

The association of plaque and gingivitis with fear of contamination in the current study may be explained by the increased fears which can make people pay greater attention to their health; thus improving their oral hygiene with lower plaque accumulation and gingivitis^36^. Compulsive checking was also associated with lower plaque levels which may be related to the obsession with cleanliness making people more likely to clean their teeth and, hence, reduce plaque accumulation^37^.

The pandemic had negative impact on the economic and financial conditions causing stress and this was associated with higher odds of reporting xerostomia. This agrees with previous studies linking stress, anxiety, depression with dry mouth and hyposalivation^38–40^. In this study, coping was significantly associated with lower odds of reporting xerostomia which may be because coping decreases stress levels leading to better oral health outcomes. However, it is important to note that the E-values for the association between xerostomia, stress and coping indicated the considerable role that unmeasured confounders such as medical conditions and medication may have in explaining away these associations.

Previous research emphasized the importance of mental and psychological wellbeing for oral health. Stress, anxiety, and depression can lead to poor oral health outcomes^41–43^. The present study adds to the literature by focusing on the association between COVID-19 specific stresses and stress-related oral conditions. These findings can clarify part of the mechanism by which the COVID-19 pandemic affects oral health, although further studies are needed for better understanding. The observed associations add to the emerging evidence about the link between oral and mental conditions and support the call for integrated planning of their care services. Future studies are needed to explore how different stresses and coping mechanisms affect other oral health conditions and how to mitigate the possible negative effects of these stress on oral health.

## Conclusion

Less plaque accumulation and gingival inflammation were associated with greater fear of COVID-19 contamination and more compulsive checking and reassurance-seeking. There were higher odds of reporting xerostomia in association with greater fear of COVID-19 socioeconomic consequences, and less coping and resilience. Our findings suggest a possible association between COVID-19 stresses and stress-related oral health conditions that needs further assessment to control for unmeasured confounders.

## Data Availability

The datasets used and/or analyzed during the current study are available from the corresponding author on reasonable request.
